# Unrecognized hand ischemia after intraarterial drug injection: successful management of a "near miss" event

**DOI:** 10.1186/1754-9493-2-32

**Published:** 2008-12-09

**Authors:** Kyros Ipaktchi, Ramin Ipaktchi, Andreas D Niederbichler, Peter M Vogt, Karsten Knobloch

**Affiliations:** 1 Department of Orthopedic Surgery, Denver Health Medical Center, University of Colorado School of Medicine, Denver, Colorado, USA; 2 Department for Plastic and Reconstructive Surgery, Hannover Medical School, Hannover, Germany

## Abstract

**Background:**

Complications arising from accidental intraarterial drug injections have been described in the past. However, given the multitude of injected substances and complex pathophysiology, guidelines regarding diagnosis and management of patients with intraarterial injections remain vague. As such it remains unclear, when to expect limb ischemia and whether and for how long to monitor patients after intraarterial injections.

**Case report:**

We present the case of a "near miss event" in an i.v. drug abuser presenting to the emergency department 3 hours after injection of water dissolved zolpidem (Ambien™) tablets into the right ulnar artery. Chief complaint was forearm pain. Clinical examination at the time revealed no concern for limb ischemia and patient was discharged. The patient returned unplanned 18 hours after injection with an ischemic right hand. Angiography revealed no flow in the distal ulnar artery and minimal flow in the palmar arch. Emergent intraarterial thrombolysis with Urokinase was performed and restored hand perfusion. Clinical follow-up 3 months after injury showed full recovery with regular recapillarisation and normal Allen test.

**Conclusion:**

This case report highlights the need to rigorously monitor patients with suspected intraarterial injections for potential delayed onset of limb ischemia. This is to our knowledge the first described case report of a successful revascularization after prolonged ischemia with delayed onset after zolpidem injection. We recommend close monitoring of these patients for at least 24 hours in addition to starting prophylactic anticoagulation.

## Background

Accidental intraarterial drug injections can occur as an iatrogenic complication or – as seen increasingly in emergency rooms worldwide – as a result of attempted intravenous injections in drug abusers [1,2]. The majorities of these latter cases are encountered in the upper extremity, and affect mostly the radial and cubital arteries due to easier accessibility and proximity to major target veins such as the cephalic and basilic vein [3,4]. Distally located injection sites, such as the one described in this case report have compared to injection into larger caliber vessels (e.g. femoral artery) a higher risk of inducing limb ischemia [5]. The advent of ischemia in an extremity however has a multifactorial origin and can be dependent on site of injection, the physicochemical drug properties and volume of the injected drug. Furthermore, on the patient side, various different pathophysiological responses, ranging from vasospasm, thrombosis, acral embolism due to non dissolvable drug components or thromboembolism can be encountered. This reflects the difficulty in treating this group of patients and the fact that there are no current universal evidence-based guidelines regarding the management of inadvertent intraarterial drug injections [3,4]. This case report describes a "near miss" event in a patient presenting with forearm pain but no signs of apparent ischemia in the emergency room after intraarterial injection of tap water dissolved zolpidem (Ambien™) tablets – an imidazopyridine drug commonly prescribed for short-term treatment of insomnia. The patient returned the next day with an ischemic hand necessitating urgent and fortunately successful thrombolysis for limb salvage. This case report highlights the importance of maintaining a high level of suspicion for delayed onset of ischemic complications in patients with accidental intraarterial drug injection. Intraarterial injection of dissolved crushed tablet preparations appear to have a higher risk for inducing ischemia and authors recommend close monitoring of these patients for at least 24 hours.

## Case presentation

**Figure 1:**
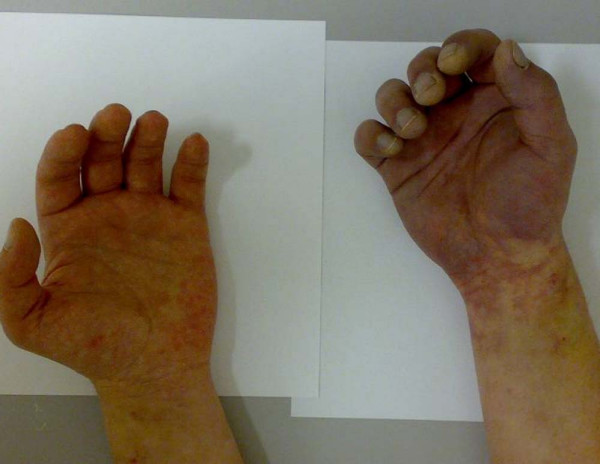
Presentation at 18 hours post injection: Significant bluish discoloration of ischemic right hand distal to wrist flexion. Hypoesthesia distal to wrist crease. Pathological Allen's test.

**Figure 2:**
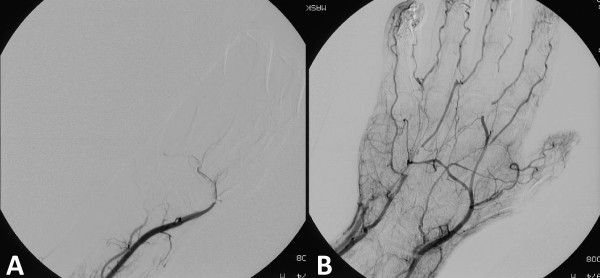
**A/B**. **A**. Angiographic study of right hand showing absent contrast flow in distal ulnar artery and deep palmar arch, incomplete filling of superficial palmar arch, hypoperfusion of digits 1 and 2 and no detectable flow in digits 3 to 5. **B**. Angiographic study of right hand 6 hours after thrombolysis with intraarterial Urokinase: Restoration of perfusion in distal ulnar artery and right hand.

A 33 year old right handed male i.v. drug abuser presents to the emergency department three hours after reportedly self injecting water dissolved crushed zolpidem tablets into the right forearm. His chief complaint was forearm tenderness extending down to the hand. The patient described an immediate onset of pain, which radiated down the arm into the hand. Clinical examination revealed a fresh injection mark at the ulnar side of the distal right forearm without signs of local inflammation. The patient had a neurovascular intact hand with 5/5 motor strength for wrist and finger extension and flexion. Digital two point discrimination, as well as capillary refill of acral digits was found to be within normal limits and a strong radial pulse was palpated, no ulnar pulse was documented. No further apparative diagnostic were performed at initial presentation. The patient was discharged with analgesic medication and told to return if symptoms aggravated. 18 hours after the injection the patient was urgently referred back to the ED by his general practitioner and presented with an ischemic hand. The right hand was found to be cold and held in a flexed position. There was absent ulnar artery pulse and only a faint radial artery pulse palpable, Allen's test was pathological. The hand showed blue discoloration and there was tenderness over the thenar eminence and hypoesthesia over the digits 1 through 5 as evidenced by a two point discrimination of greater than 8 mm (See Fig 1). Emergent angiography revealed no flow in the distal ulnar artery and absent deep and superficial palmar arch perfusion (Fig. 2a). The patient was admitted to the intensive care unit and urokinase lysis via an indwelling intraarterial catheter was started at 20 hours after injection with 240.000 IU/h over 2 hours followed by half the dose for 2 hours.

**Figure 3:**
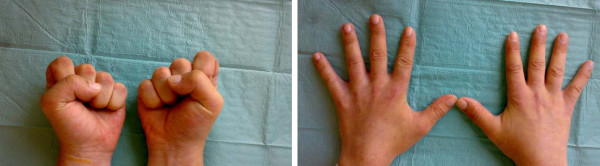
Follow up at 3 months post trauma: Full functional recovery of right hand function.

The patient was closely monitored for possible compartment syndrome of the forearm and hand with potential need for decompressive surgical intervention. Within 3 hours after start of thrombolytic therapy there was a return of normal skin color to the right hand and restoration of a strong Doppler detectable ulnar artery and palmar arch pulse signal. Clinically, the Allen test turned normal. There was no need for compartment decompression and follow-up angiography the next day (Fig. 2b) demonstrated restoration of ulnar as well as digital artery flow with normal Allen test. Patient was continued on i.v. heparin until discharge at day 6 after trauma. In addition he was placed on 100 mg aspirin/day for 2 weeks. At discharge he had regained full motor strength of finger flexion and extension as well as hand intrinsics with the exception of a 4/5 strength of thumb opposition. A follow-up at 3 months showed bilateral equal skin coloration, no trophic changes to skin and skin appendages of the right hand, normal Allen test and an intact neurovascular exam with 5/5 motor function throughout and intact sensation (Fig 3).

## Discussion

Intraarterial injections in the extremities can have devastating consequences such as limb ischemia and limb loss[3]. In contrast to iatrogenic inadvertent injections, intraarterial injections in i.v. drug abusers are often complicated by delayed presentation, a potentially occult i.v. drug abuse history and a wider array of injected substances, some of which – as in our case – were not designed to be injected in the first place [1,6,7]. Increased incidence in injection of crushed oral formulations is explained by easier drug availability and lower costs and is associated with more complications[8]. The multitude of clinical presentations and time course of the developing symptomatology can challenge emergency room physicians attending to these patients. There are only few publications to date discussing management of limb ischemia subsequent to intraarterial drug injections in i.v. drug abusers and thus, currently there are no evidence-based criteria for this entity.

The clinical work-up for all patients with suspected intraarterial drug injections must include a thorough history, including identification of injected drug, site of injection and onset of symptoms. Typically upon intraarterial injections, patients can experience immediate painfulness and tenderness extending from the site of injection to the hand – a symptom commonly described as a "hand trip"[9]. This should raise a red flag to the possibility of an intraarterial drug application. It is crucial to attempt drug identification, which can be difficult in occult i.v. drug abuse, as injections of crushed, legally available drugs lends itself to drug abuse in patients appearing "free" of illicit i.v. drug abuse history[1].

Diagnostic evaluation must include manual (Allen test) and Doppler-based examination of extremity pulses including palmar arch and digital pulses. This said, the presence of a detectable pulse at the wrist cannot rule out a proximal based vascular occlusion. Here ankle brachial index measurements or arterial pressure index measurements (API) can strengthen the suspicion for proximal occlusion. The API is performed by placing a blood pressure cuff just above the wrist of the injured limb and the systolic pressure is determined by Doppler probe at the distal forearm pulse sites for radial or ulnar artery. The identical measurement is performed on the uninjured limb and the API is calculated by dividing systolic pressure in the injured limb by the systolic pressure in the uninjured limb. This tool has been validated in the setting of penetrating as well as blunt injuries [10-12] In a study of mixed mechanism injuries (blunt and penetrating), it was found that an API < 0.9, had a sensitivity and specificity of 95% for major arterial injury[11]. Currently, angiography is still the gold standard for detection of vascular pathologies in the setting of inadvertent arterial injections and allows for interventional therapy. Besides, the role of CT angiography, MRI angiography or laser Doppler flowmetry in this application still has to be determined. A comparative study found a high degree of comparability between angiographic findings and color coded duplex sonography in ischemic digits[13]. Any signs of limb ischemia must alert the clinician to closely monitor for compartment syndrome.

Ischemic states can occur based on distinct pathologies which need to be identified to guide therapy. Drug-sided factors increasing the risk for vascular complications can be attributed to physicochemical, pharmacological and galenic-formulation properties of the injected drug. As such very acidic or hyperosmolar substances can result in direct tissue necrosis at the site of injection with obliteration of vascular flow, as has been described in injections of dissolved flunitrazepam tablets[14]. Of all pharmacological properties, vasoconstrictive effects are in the scenario of intraarterial injection the most problematic. Here sympathomimetics such as oxymethazoline, cocaine and methylphenidate can result in sustained vasospasm and tissue malperfusion [15,16].

The galenic formulation of a drug can induce ischemic complications which can depend on the drug carrier: Here, micro-particle drug carrier as commonly seen in tablet preparations can result in non dissolving occlusions of terminal digital vasculature resulting in acral necrosis. Chang et al. described the injection of crushed zolpidem, the identical drug and form of drug administration as in our case, however in that case gangrenous loss of several digits was seen, despite aggressive therapeutic management and was attributed by the authors to microcrystalline cellulose, the drug carrier in zolpidem [17].

On the patient-side, any arterial puncture can induce prolonged vasospasm due to local endothelial trauma at the site of injection – a major risk factor of radial artery cannulation which can precipitate ischemic complications [18]. Patients can present with distal, acral based ischemia as in particle embolism and alpha sympathomimetic drug injections or in more proximal based ischemia due to larger vessel thrombosis. However clinically mixed presentations predominate which can blur the clinical picture: Local thrombosis followed by thromboembolic events are commonly seen and are ascribed by many authors to be the common converging route leading to ischemia in all inadvertent arterial injections [3].

Depending on the clinical picture and drug injected, the therapeutic management aims at restoring flow in ischemic limbs, preventing thromboembolic complications and preserve hand function by avoiding secondary muscle ischemia and necrosis through compartment syndrome [5,19]. Where appropriate, antagonizing vasospastic drug effects should be instituted via systemic or local intraarterial alphablockade or phosphodiesterase inhibition with agents such as phentolamin or papaverin [20]. The role of phosphodiesterase inhibitors such as sildenafil has to be determined in this regard in the future. Treatment includes anticoagulation using i.v. heparin to prevent the propagation of thromboembolic complications and early lysis of thrombotic arterial occlusions must be instituted either via interventional radiology, or intraoperatively [21-23]. The role of low-molecular weight heparins in contrast to intravenous heparin has not been addressed thus far. Successful salvage of ischemic states in this context has been also described for the combination of PGE (1) and local fibrinolytic therapy with rt-PA [24].

Traditionally operative embolectomy has been successfully described for occlusions proximal to the midforearm [25,26]. Although digital artery microembolectomy has been described, this remains a not universally advocated technique[27]. Considering the thromboembolic pathophysiology of this injury and the anatomy of hand vasculature with branching endarterial digital distribution, most authors appear to favor thrombolytic therapy [28,29]. Unlike in the lower extremity there exists no larger size comparative study regarding surgery versus lysis [30]. Although the Cochrane review for lower extremity ischemia showed successful limb salvage after more than 24 hours with thrombolytic therapy, we present to our knowledge the first case describing a successful revascularization in intraarterial injection of crushed zolpidem after more than 18 hours in the upper extremity.

## Conclusion

The rising popularity in injection of crushed water dissolved tablet formulations has resulted in increased incidence of acute upper extremity ischemia seen in emergency rooms. The onset of hand ischemia can be – as seen in our case report – delayed and the risk of pending limb loss may be missed upon initial presentation. We describe the successful thrombolytic management of an ischemic hand in a patient presenting 18 hours after accidental intraarterial zolpidem injection into the ulnar artery and an initial bland, innocuous appearance 3 hours after the injury. Physicians attending primarily to these patients must be aware to this pathology and authors recommend 24 hours of close observation even in patients appearing to have a well perfused extremity. Additionally we recommend heparinization to reduce the risk for thromboembolic complications.

## Consent

Written informed consent was obtained from the patient for publication of this case report. A copy of the written consent is available for review by the Editor-in-Chief of this journal

## Competing interests

The authors declare that they have no competing interests.

## Authors' contributions

KI, manuscript draft, RI: Case provision and manuscript revision, PMV, ADN and KK: manuscript revision.
